# Indirect Energy Flows in Niche Model Food Webs: Effects of Size and Connectance

**DOI:** 10.1371/journal.pone.0137829

**Published:** 2015-10-05

**Authors:** Jane Shevtsov, Rosalyn Rael

**Affiliations:** 1 Division of Life Sciences, UCLA, Los Angeles, CA, United States of America; 2 Tulane-Xavier Center for Bioenvironmental Research, Tulane University, New Orleans, LA, United States of America; Vrije Universiteit, NETHERLANDS

## Abstract

Indirect interactions between species have long been of interest to ecologists. One such interaction type takes place when energy or materials flow via one or more intermediate species between two species with a direct predator-prey relationship. Previous work has shown that, although each such flow is small, their great number makes them important in ecosystems. A new network analysis method, dynamic environ approximation, was used to quantify the fraction of energy flowing from prey to predator over paths of length greater than 1 (flow indirectness or FI) in a commonly studied food web model. Web structure was created using the niche model and dynamics followed the Yodzis-Innes model. The effect of food web size (10 to 40 species) and connectance (0.1 to 0.48) on FI was examined. For each of 250 model realizations run for each pair of size and connectance values, the FI of every predator-prey interaction in the model was computed and then averaged over the whole network. A classification and regression tree (CART) analysis was then used to find the best predictors of FI. The mean FI of the model food webs is 0.092, with a standard deviation of 0.0279. It tends to increase with system size but peaks at intermediate connectance levels. Of 27 potential predictor variables, only five (mean path length, dominant eigenvalue of the adjacency matrix, connectance, mean trophic level and fraction of species belonging to intermediate trophic levels) were selected by the CART algorithm as best accounting for variation in the data; mean path length and the dominant eigenvalue of the adjacency matrix were dominant.

## Introduction

Food webs are icons of complexity, depicting intricate networks of feeding interactions. Since food webs can be studied both from the point of view of population dynamics and that of matter and energy flows, they bridge community and ecosystem ecology. Moreover, their study has led to insights that apply to other complex systems [1–3].

**Fig 1:**
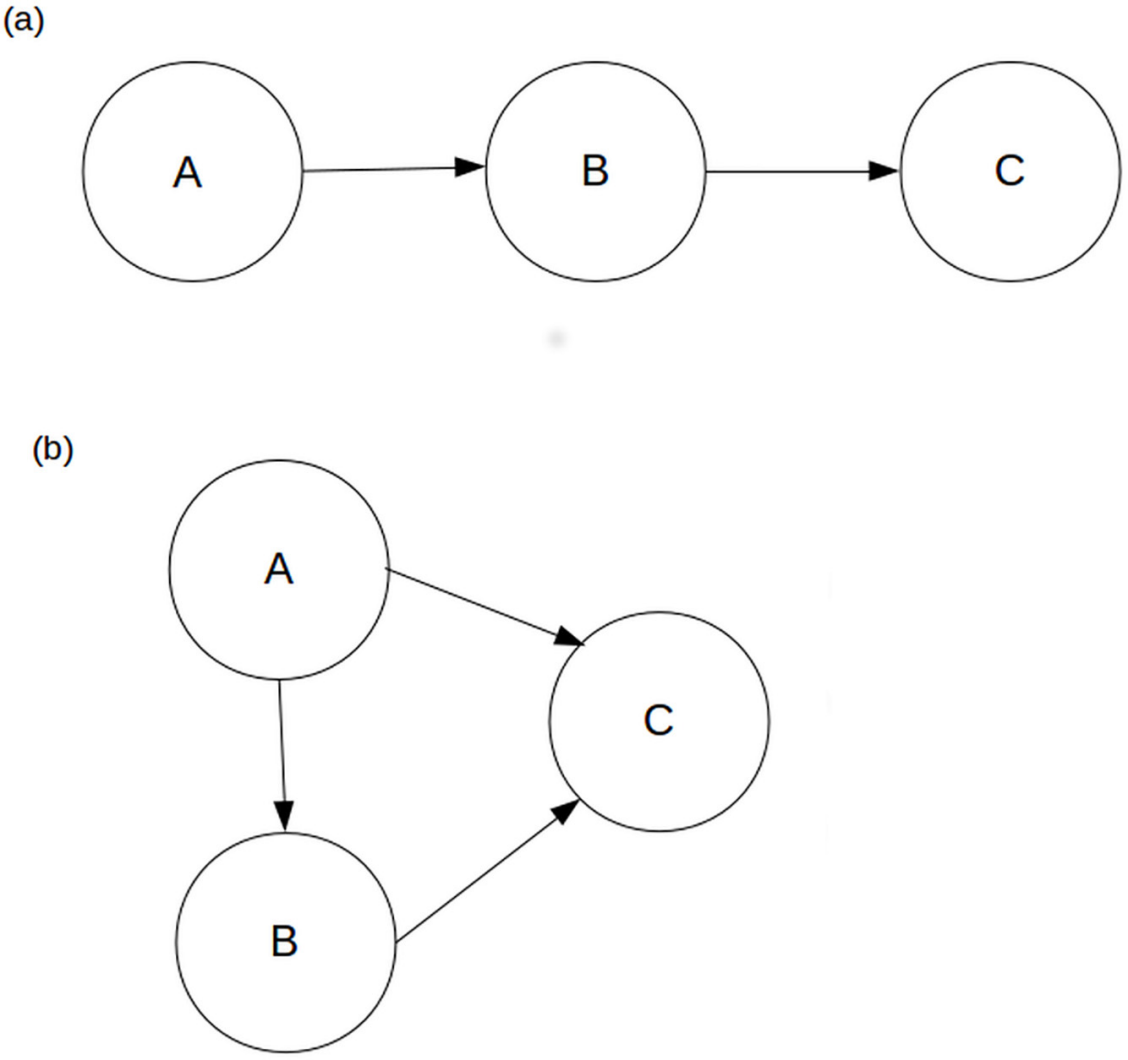
Direct and indirect flows. In the module shown in Fig 1a, species A and C are linked by both direct and indirect flows, while in the module shown in Fig 1b, they are linked exclusively by indirect flows.

Examining food webs reveals a wide variety of indirect interactions, such as indirect matter and energy flows, trophic cascades, apparent competition, indirect mutualism and commensalism, and exploitative competition [4]. Indirect flows take place when energy or nutrients move between two species by a path, termed an indirect path, that includes one or more intermediate species (Fig 1). Previous work has shown that, although individual indirect flows may be small, their great number makes them important in ecosystems. In fact, in many empirically-based ecosystem models, the fraction of total energy flow that travels over indirect paths (flow indirectness or FI) is greater than 50%, a property often described as “dominance of indirect effects” [5–9]. This high flow indirectness value implies that pairwise interactions between compartments in these systems are strongly mediated by the rest of the system.

The mathematical and conceptual framework that allows flow indirectness and many other network properties to be quantified, termed environ analysis [8, 10–12], has not previously been applied to theoretical food web models with structures similar to those of field webs and empirically-based dynamics. Most studies of indirect matter and energy flows have focused on small, highly aggregated ecosystem models [7, 9, 13], although some have looked at large, highly simplified, theoretical models [9] and steady-state empirical models of various sizes [14].

This study investigates the importance of indirect energy flow in food webs by measuring the flow indirectness of theoretical food web models and examining how it is affected by web size and connectance, defined as the fraction of possible directed links that actually exist. These variables were chosen because they are fundamental to food web research, both because they can be manipulated directly in simulations and because they directly parametrize common food web models [15–17]. Our goal is not to provide a comprehensive examination of flow indirectness in various ecological models but simply to measure it in one commonly studied model and demonstrate the potential usefulness of environ analysis and DEA. The models studied here use the niche model [16] for structure and the *n*-species Yodzis-Innes model [18, 19] for dynamics. The niche model assumes that species in a community can be ordered along a “niche” dimension [20], such that consumers mainly feed on species with a lower niche value than their own but may also feed on those with a higher niche value. The niche value is correlated with, but not identical to, body size [16, 21, 22]. Each species feeds on all species (including, potentially, its own) whose niche value lies within a specified range. For species *i* with niche value *n*_*i*_, the feeding range has width *r*_*i*_ and can be centered anywhere in the interval [*r*_*i*_/2, *n*_*i*_] [16].

The *n*-species Yodzis-Innes model uses consumer functional responses and the scaling of metabolic rate with body size [23–25] to add realism to a simple model of trophic dynamics. (Since, as described below, the analysis used to quantify flow indirectness requires a conservative currency, the model’s state variables were taken to be the total energy content of each species.) Including a functional response that saturates at high prey density improves model realism by acknowledging the fact that there is a limit to how much food an individual can consume. The use of scaling relationships helps incorporate biologically reasonable sets of parameter values into a theoretical model. The model, which employs variables and parameters whose dimensions and values are listed in Table 1, is described below. In keeping with environ analysis convention, we consider energy to flow from column *j* to row *i*, not the other way around, as is the convention in dynamic food web modeling.

**Table 1:** Parameters and variables of *n*-species Yodzis-Innes model where *m*_*i*_ is the body mass of species *i*. Parameter values were taken from [26].

Quantity	Meaning	Value used
*B*_*i*_	energy content of compartment *i*	
*r*_*j*_	intrinsic growth rate	1 for producers, 0 otherwise
*K*_*j*_	carrying capacity for producers	1
*x*_*i*_	body mass-specific metabolic rate relative to maximum producer growth rate	0.138 for producers, $0.314m_{i}^{-1/4}$ otherwise
*y*_*ij*_	consumption rate for *i* consuming *j* normalized by metabolic rate of *i*	8
*e*_*ij*_	conversion efficiency for *i* consuming *j*	0.45 for feeding on producers, 0.85 otherwise
*q*	reward sensitivity in Holling Type III functional response	1
*B*_0_	half-saturation density in functional response	0.5
*c*	strength of predator interference in functional response	1

In the absence of consumers, producer *j* grows logistically at rate $r_{j}B_{j}\left(1-\frac{B_{j}}{K_{j}}\right)$, where *B*_*j*_ is the total energy content (or population biomass) of species *j*, *r*_*j*_ is its maximum growth rate and *K*_*j*_ is the environment’s carrying capacity for species *j*. To obtain the rate at which species *j* is eaten by species *i*, we reason as follows. The rate of consumption of *j* by *i* is proportional to the population size of *i*, *B*_*i*_. The quantity *y*_*ij*_ is the maximum rate at which species *i* can consume species *j*, divided by *i*’s metabolic rate, *x*_*i*_. Multiplying this quantity by *x*_*i*_ gives *x*_*i*_ *y*_*ij*_, the maximum per-capita consumption rate for *i* preying on *j*. The functional response, *F*_*ij*_(*B*), gives the consumption rate as a fraction of this maximum rate, yielding *x*_*i*_ *y*_*ij*_ *F*_*ij*_(*B*)*B*_*i*_ for the actual rate. However, the predator does not ingest and assimilate all the prey it captures, so its consumption rate must increase to compensate for this. Dividing the previously obtained rate by the predator’s efficiency, *e*_*ij*_, accomplishes this, giving the expression *x*_*i*_ *y*_*ij*_ *F*_*ij*_(*B*)*B*_*i*_/*e*_*ij*_.

We now turn to the functional response. Following [26], a sigmoidal (Holling Type III) functional response with predator interference [27] was chosen, in part because it stabilizes the dynamics of food web models [28]. In this model, the consumer’s search rate is proportional to prey abundance raised to the non-negative power *q* [29], and consumers of a given species interfere with each other with strength *c* [27]. As a result,

$$F_{ij}\left(B\right)=\frac{B_{i}^{1+q}}{B_{0}^{1+q}+cB_{0}^{1+q}B_{j}+\sum_{k=prey}\;B_{k}^{1+q}}.$$ (1)

Following [26], the values *q* = 1 and *c* = 1 were used. (Table 1) This results in relatively high predator interference and a pronounced sigmoid functional response. The overall differential equation for producer species *j* is:

$$\frac{dB_{j}}{dt}=r_{j}B_{j}\;(1-\frac{B_{j}}{K_{j}})-\sum_{i=predators}x_{i}y_{ij}B_{i}F_{ij}\left(B\right)/e_{ij}.$$ (2)

Consumers of species *i* lose energy to metabolism at rate *x*_*i*_ *B*_*i*_, gain it from prey item *j* at rate *x*_*i*_ *B*_*i*_ *y*_*ij*_ *F*_*ij*_(*B*), and lose it to consumers of species *k* at rate *x*_*k*_ *y*_*ki*_ *B*_*k*_ *F*_*ki*_(*B*)/*e*_*ki*_ [19]. Overall, we have:

$$\frac{dB_{i}}{dt}=-x_{i}B_{i}+x_{i}B_{i}\sum_{j=prey}y_{ij}F_{ij}\left(B\right)-\sum_{k=predators}x_{k}y_{ki}B_{k}F_{ki}\left(B\right)/e_{ki}.$$ (3)

Table 1 summarizes the model’s parameters and their values.

To parametrize the model, empirically documented relationships between trophic level and body mass [30] were used to assign body masses to species in the model. Following [31], the (generally non-integer) trophic level of each species was computed as the mean of two quantities: (1) the integer distance between the target species and the closest basal species (those that do not prey on any other species); and (2) Levine’s [32] generally non-integer flow-based trophic position, computed under the assumption that predators receive equal fractions of their diet from all prey species. The equal flows assumption allows flow-based trophic positions to be assigned to species in a purely topological web. The expression for flow-based trophic position is:

$$TL_{i}=1+\sum_{j=1}^{S}TL_{j}p_{ji}$$ (4)

where *TL*_*i*_ is the trophic level of species *i*, *S* is the total number of species in the food web, and *p*_*ji*_ is the fraction of species *i*’s diet provided by species *j*. The mean of this quantity and distance from a basal species was used because it can be computed from topological information and provides a close approximation to the true flow-based trophic position in food webs for which flow data is available [31]. Species were then assigned metabolic rates using the 3/4-power scaling relationship between metabolic rate and body size [23–25].

A new flow-based dynamic network analysis method called dynamic environ approximation (DEA [33]) was used to compute FI. The basic logic of DEA is as follows. If a food web has adjacency matrix **A**, then **A** ^*k*^ gives the number of paths of length *k* between each pair of species and $\sum_{k=1}^{m}A^{k}$ gives the total number of paths of length *m* or less between each such pair [34, 35]. If the structure of the network changed over time, then the number of paths would be given by the product series **A**(*t*) + **A**(*t*)**A**(*t* + 1) + … + (**A**(*t*)**A**(*t* + 1)…**A**(*t* + *m*)). DEA uses a related product series of matrices describing energy flows in the food web to trace the flows through the system. The flow matrix is then normalized by the total outflow from the donor species to create a matrix, **G**, of nondimensional flow intensities for each integer time step. Then, for a window of *m* time steps, we have integral flow **N**(*t*) = **I** + **G**(*t*) + **G**(*t*)**G**(*t* + 1) + … + (**G**(*t*)**G**(*t* + 1)…**G**(*t* + *m*)), where **N**(*t*) is the matrix transforming inputs received at time *t* into eventual flows throughout the system, summing over all times up to *t* + *m*. Flow indirectness is then computed as *FI*_*ij*_(*t*) = (*N*_*ij*_(*t*) − *G*_*ij*_(*t*))/*N*_*ij*_(*t*) [33]. This method was used to find FI for each interaction in the web and the entries of the **FI**(*t*) matrix were then averaged.

## Results

**Fig 2:**
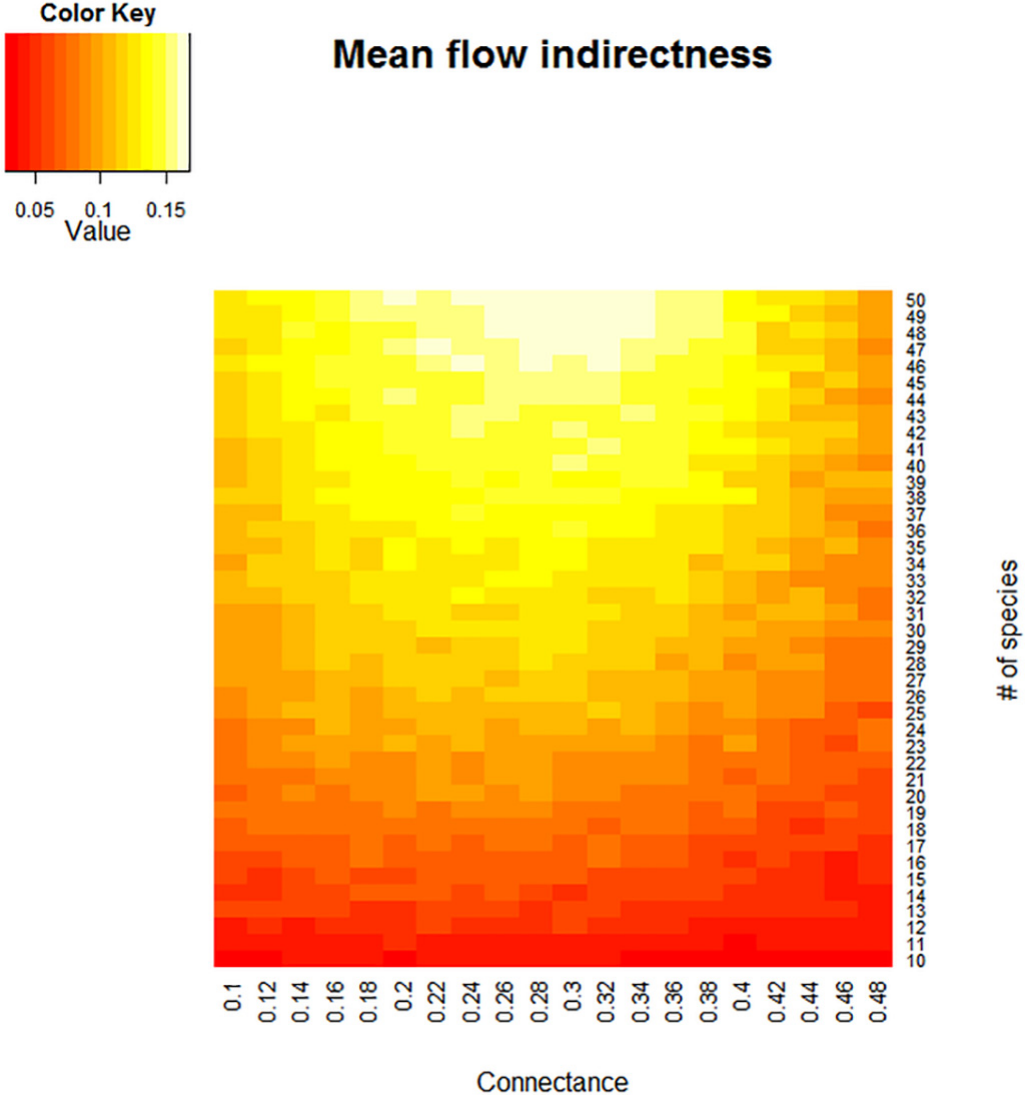
Mean flow indirectness as a function of nominal system size and connectance.

Over the full range of parameter values, the mean flow indirectness of the model food webs was 0.092, with a standard deviation of 0.0279. It increased with system size but peaked at intermediate connectance levels, resulting in the pattern seen in Fig 2.

### Determinants of flow indirectness

A classification and regression tree (CART) analysis performed in R [36] with the package rpart [37] was used to explore which aspects of web structure were most strongly correlated with mean FI. The algorithm performed a split only when doing so increased *R* ^2^ by at least 0.01. Also, to avoid overfitting, 10 cross-validations were performed at each step.

**Fig 3:**
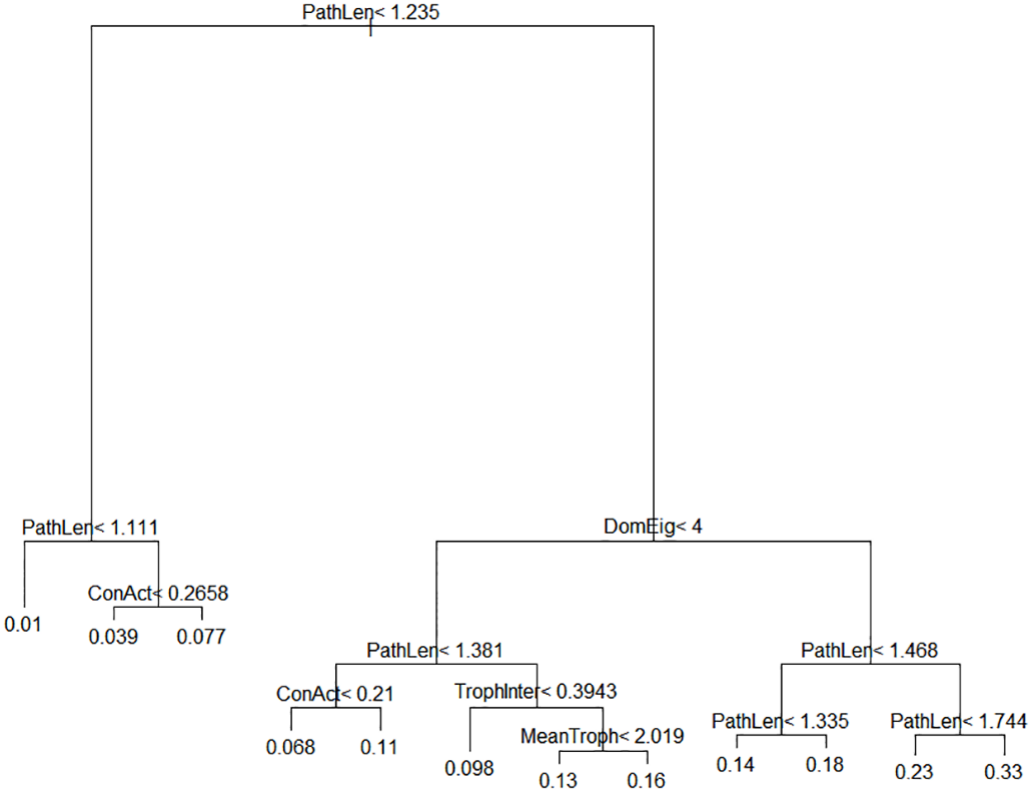
Classification and regression tree for flow indirectness. If the node condition is met, the left-hand branch is taken; otherwise, the right-hand branch is taken. Numbers at branch tips are predicted FI values. Key to variables: PathLen—mean path length; DomEig—dominant eigenvalue of adjacency matrix; ConAct—actual connectance; TrophInter—fraction of species with both predators and prey; MeanTroph—mean trophic level.

**Fig 4:**
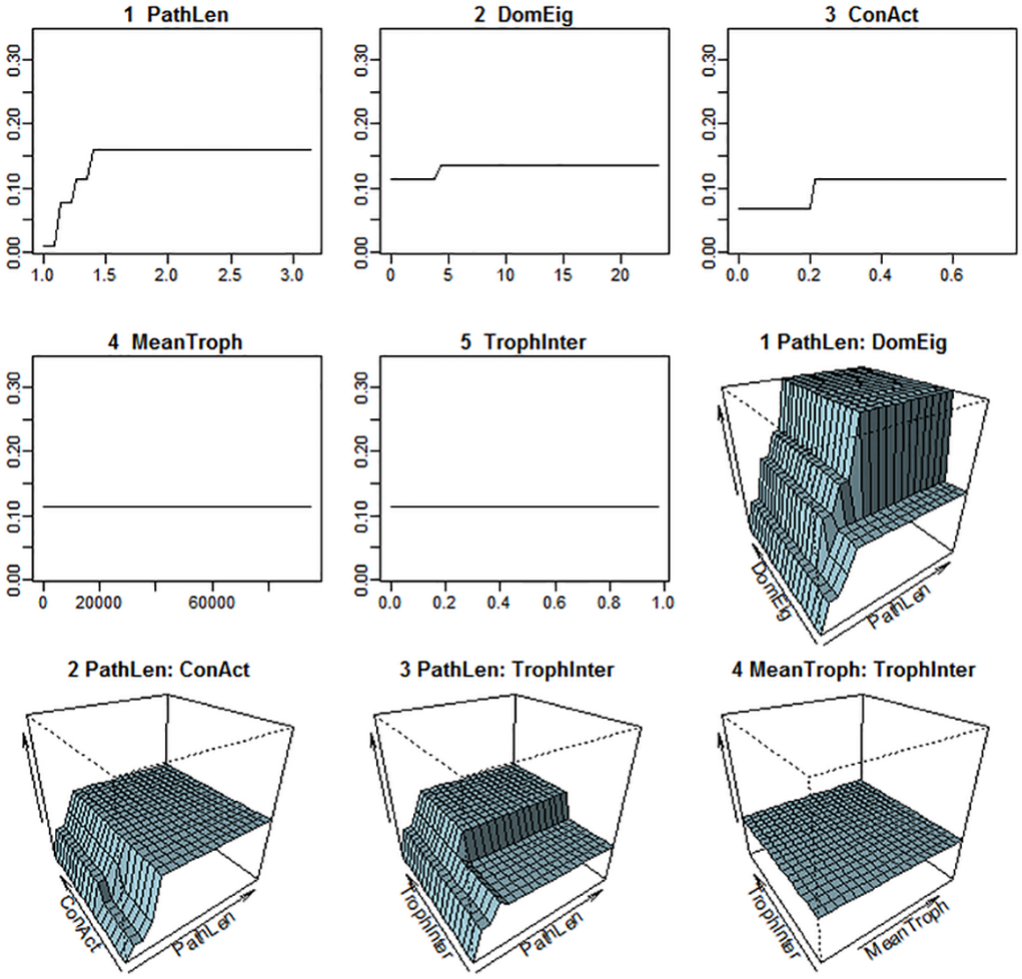
Partial dependence plots showing the relationship between FI values and the predictor variables in the CART analysis. Key to variables: PathLen—mean path length; DomEig—dominant eigenvalue of adjacency matrix; ConAct—actual connectance; TrophInter—fraction of species with both predators and prey; MeanTroph—mean trophic level.

Out of 27 potential ecological and graph-theoretic predictor variables, only five (mean path length, dominant eigenvalue of the adjacency matrix, connectance, mean trophic level and fraction of species belonging to intermediate trophic levels) were selected by the CART algorithm as best accounting for variation in the data. Two of these, mean path length and dominant eigenvalue of the adjacency matrix, were dominant. (Fig 3) The CART model accounted for 82.2% of the variation in FI. A full list and explanation of the potential predictor variables used is given in the Supporting Information. Fig 4 provides a more detailed look at the relationship between flow indirectness and these predictor variables.

Despite predicting the observed FI values very well, the CART model does not explain the curvilinear relationship between connectance and FI. A quadratic model was therefore used to test the relationship between connectance and path length. The model had an *R* ^2^ of 0.9639 and the Δ*AIC* between this model and a linear model was -240817. (In both models, the intercept was forced through zero, as no other value is possible.)

### Application to field data

**Fig 5:**
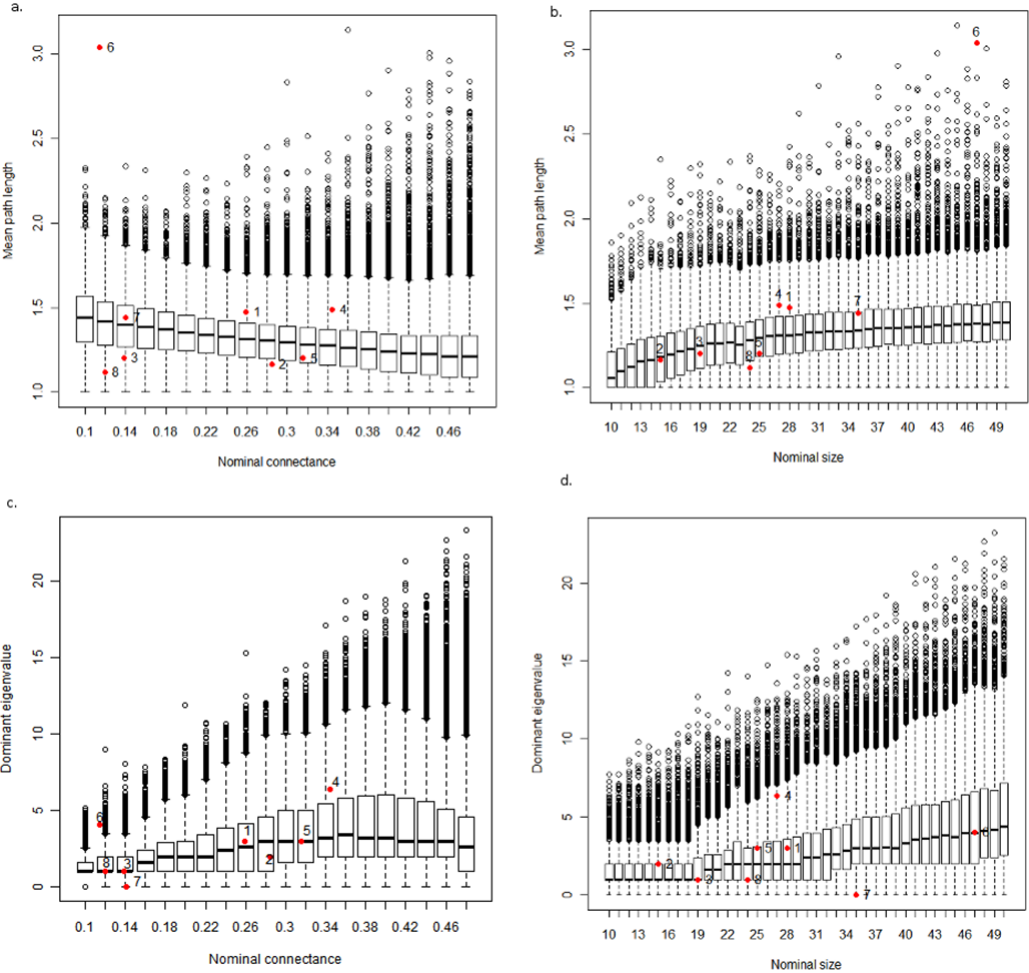
Model food web mean path length and dominant eigenvalue as a function of nominal size and connectance. Each boxplot shows the median, 75th and 25th percentiles for each data set, with whiskers extending up to 1.5 times the interquartile range and any points falling outside that area being plotted individually. In (a) and (c), mean path length generally decreases with connectance, while the dominant eigenvalue shows a curvilinear pattern. In (b) and (d), both mean path length and dominant eigenvalue increase with web size. Together, these relationships largely account for the patterns in Fig 2. Red numbered dots show where real food webs fall on these graphs. Key: 1. Benguela ecosystem; 2. Bridge Brook Lake; 3. Canton Creek; 4. Coachella Valley; 5. Skipwith Pond; 6. St. Marks Seagrass; 7. St. Martin Island; 8. Stony Stream p.

The simulation results were compared to eight commonly studied topological food webs ([38–43]) whose sizes and connectances fell within the range of the parameter scan. As Fig 5 shows, most of the relationships among food web size, connectance, mean path length and dominant eigenvalue in empirical food webs fall well within the range of model webs. The major exceptions are the St. Marks seagrass web, which has a much larger mean path length for its size and connectance than any of the other modeled or empirical webs, and the St. Martin Island web, whose dominant eigenvalue is zero because the web contains no cycles.

Predictions of the flow indirectness values of the empirical webs were made by following the CART tree developed using the simulated webs. (Table 2) Freshwater food webs had consistently lower predicted FI values than terrestrial and marine webs. This is due to the freshwater webs’ lower mean path lengths, which appear to simply be a result of their smaller sizes. Size-normalized mean path length did not vary systematically with ecosystem type.

**Table 2:** Network characteristics and predicted flow indirectness values for empirical food webs. Predictions were made by following the CART tree in Fig 3.

Ecosystem	Size	Connectance	Dominant eigenvalue	Mean path length	Intermediate fraction	Mean trophic level	Predicted FI
*Freshwater*							
Bridge Brook Lake [38]	15	0.284	2.00	1.16	0.93	2.35	0.077
Canton Creek [39]	19	0.139	1.00	1.20	0.74	2.12	0.039
Skipwith Pond [40]	25	0.315	3.00	1.20	0.92	2.67	0.077
Stony Stream [39]	24	0.12	1.00	1.12	0.75	2.25	0.039
*Marine*							
Benguela Current [41]	28	0.259	3.00	1.47	0.96	3.18	0.16
St. Marks Seagrass [42]	47	0.115	4.03	3.04	0.87	3.52	0.33
*Terrestrial*							
Coachella Valley [47]	27	0.344	6.35	1.49	0.96	3.00	0.23
St. Martin Island [43]	35	0.140	0.00	1.44	0.77	2.62	0.16

## Discussion

The flow indirectness of a food web provides an indication of the extent to which pairwise interactions are mediated by the network in which they are embedded. When FI is high, a significant portion of the effect of an experimental manipulation of the abundance of one species on that of another will be determined by the rest of the food web and may not generalize to other systems.

We found that the mean flow indirectness over the full range of size and connectance values was 0.092, with various combinations of topological variables giving FI values as low as 0.01 or as high as 0.33 (Fig 3). The increase of FI with web size (Fig 2) is particularly important, as it indicates that, in real food webs, a substantial proportion of energy can be expected to travel over indirect paths (Table 2). Due to dissipation, each individual path carries little energy, but the number of paths makes up for this [6]. Cycling plays an important role in pathway proliferation, so it makes sense that the dominant eigenvalue of the network, a measure of the amount of cycling in the network [44, 45], is strongly positively related to FI.

The quadratic relationship between connectance and path length explains the curvilinear relationship seen in Fig 2. It may be that increasing connectance first results in longer paths by connecting more species, but then the increasing number of links short-circuits long paths, resulting in a lower mean path length and correspondingly lower flow indirectness.

It will be useful to examine the effects of other system attributes on the flow indirectness and find out whether this quantity is linked to the vulnerability of food webs to species loss. [46] found that, for sixteen empirical topological food webs, vulnerability to cascading extinctions in the face of species loss was negatively correlated with connectance and uncorrelated with the prevalence of omnivory (in spite of the correlation between omnivory and connectance). However, the webs examined in that study had connectances ranging from 0.026 to 0.315—values falling within the range in which flow indirectness increases with connectance. (Fig 2) It would be instructive to examine the effects of species loss on model webs with higher connectance values, to see whether the positive relationship between connectance and robustness continues to hold and whether omnivory becomes a more important determinant of robustness as flow indirectness declines.

Two major frameworks exist for studying networks of trophic interactions and the movement of energy within ecosystems: those of community and ecosystem ecology. When food webs are studied from a community ecology perspective, the emphasis is on individual species and their population dynamics. Such webs are as detailed as possible but often omit parts of the biota at the study location, especially decomposers and detritivores in terrestrial systems. (The Coachella Valley, CA food web of [47] is a prominent exception.) By contrast, the ecosystem framework uses comprehensive, usually highly aggregated models that focus on the movement of energy and nutrients. Researchers working within these two frameworks have ignored each other’s work to a remarkable extent.

Environ analysis [8, 10, 11] is a set of conceptual and mathematical tools for analyzing networks of stocks and flows. It has traditionally been applied to phenomenological models of real ecosystems. This is both a strength and a weakness, in that the analysis stays close to reality but is tied to a relatively small number of models that are usually highly aggregated. In particular, the six-compartment intertidal oyster reef model of [48] may be the *Drosophila* of environ analysis because of the number of techniques and hypotheses that have been demonstrated and tested using it [13, 33, 49–51].

This study is not the first to apply environ analysis to a large synthetic model. [9] created models of ecosystems by assigning species to one of six functional groups: primary producers, herbivores, carnivores, omnivores, detrital feeders and detritus. Each functional group, including detritus, contained the same number of species, ranging from five to one hundred. Biologically plausible intergroup interactions were then randomly assigned. The model used linear donor-controlled dynamics with randomly selected coefficients. Thus, this model possessed some realism with regard to functional groups, very little with regard to network structure, and almost none (except in the case of flows to detritus) with regard to dynamics.

The advantage of theoretical models such as the Yodzis-Innes model is that they describe causal relationships between species. Compartment models, on the other hand, are typically phenomenological, “bookkeeping” models. When dynamical assumptions such as donor control are added to these models, they are typically very simple and lack biological justification. The relatively detailed causal assumptions and parameter constraints of the Yodzis-Innes model may be criticized as being overly complex and unrealistic, but they are probably less wrong than linear models with donor control, which assumes that the amount of prey eaten by a predator species depends only on the prey’s population size. However, research on such dynamically simple models has produced insights into ecosystem function and network properties.

Working within the framework of linear steady-state models of conservative energy and matter flows and storages, [49] identified six network characteristics that directly increase flow indirectness in compartment models: number of compartments, connectance, storage, cycling, feedback and magnitude of direct flows. Most of the model webs in this study had much lower mean flow indirectness values than the ecosystem models examined in previous work [9, 14]. This is likely due to the fact that niche model webs, unlike the models studied before, do not include detritus or detritivores. Therefore, they contain substantially less cycling than ecosystem-oriented models. Since cycling greatly increases the fraction of model currency traversing indirect paths [49], its absence must reduce flow indirectness.

This omission is significant because detritivory is a nearly universal feature of real food webs [52–54]. Thus, the current results almost certainly underestimate the true importance of indirect flows in natural food webs and future work should attempt to include detritus and detritivores. A logical next research step would be to use a version of the niche model modified to include detritus and detritivory, such as that of [55].

Other important topics for future research include the sensitivity of the results described here to parameter values and model assumptions and the examination of energy cycling in model webs with and without detritus [6, 13]. In particular, the standard niche model uses niche values that are uniformly distributed between 0 and 1. However, the niche value has been found to be correlated with body size [16, 21, 22]. Therefore, the distribution of niche values should be derived from body size distributions observed in nature. The allometric diet breadth model [56] approaches this idea but relies on previously specified body size data, although this could be randomly generated. A simpler approach would retain all the assumptions of the niche model but use a more realistic distribution of niche values.

The results reported here helps bridge contemporary food web ecology and systems ecology, while providing a new way of looking at ecosystem complexity. It is also possible to apply dynamic environ approximation to non-trophic stock and flow networks such as dispersal networks [57] and human systems such as roads and economies, and doing so may provide insights into their function.

## Methods

### Web Construction and Simulation

The goal of this study was to explore the importance of indirectness in a commonly studied theoretical food web model, the niche model [16]. This model was selected because it is frequently studied and reproduces many features of real food webs with a fair degree of accuracy [16]. In this model, each species has a niche value, *n*_*i*_, a feeding range width, *r*_*i*_, that can be interpreted as the fraction of possible niche values that can be consumed by species *i*, and a feeding range center. The niche value for each species is drawn from a uniform distribution ranging from 0 to 1. Range centers are drawn from a uniform distribution ranging from *r*_*i*_/2 to *n*_*i*_. A uniform distribution is used both for its simplicity and to reflect the hypothesis that niche values in real ecosystems are roughly uniformly distributed.

Species’ range widths are generated by drawing values from a beta distribution whose mean is twice the connectance of the web and multiplying them by the species’ niche value. (Specifically, the niche model uses a beta distribution with *α* = 1 and $\beta =\frac{1-2C}{2C}$, where *C* = 2*L*/*S* ^2^, the connectance of the web, *S* is the number of species, and *L* is the number of links.) Since the expected value of this distribution is 2*C* and that of *n*_*i*_ is 0.5, this procedure results in range width having an expected value of *C*. Because niche values are uniformly distributed on the [0, 1] interval and a consumer’s feeding range width is the fraction of this interval that contains potential prey, the fraction of species a given consumer preys on is approximately its range width. This gives the food web the desired connectance. Each species is assumed to prey on all species within its range, including itself, and a food web directed adjacency matrix is assembled [16].

Candidate webs generated by this method were tested to ensure that they had at least one producer and consisted of only one set of connected species, termed a component in graph theory [35]. For the latter test, the Laplacian matrix, **L**, was used. This matrix is defined as the difference between the degree matrix **D**, which has the degree of the graph’s nodes on the diagonal and zeros elsewhere, and the undirected adjacency matrix **A** from which self-loops are excluded, making *a*_*ii*_ = 0. The equation for the Laplacian matrix is then **L** = **D** − **A**. The number of times 0 appears as an eigenvalue of the Laplacian is the number of components in the graph [58, 59].

If a web passed these tests, trophic levels were assigned to each species as the mean of distance from the closest basal species and the flow-based trophic position method (Eq 4), computed under the assumption that predators receive equal fractions of their diet from all prey species [31, 32]. (The equal flows assumption allows trophic levels to be computed for a purely topological web.) Taking the mean of these two methods used provides a good approximation to real trophic levels in quantitative food webs and ecosystem models [31]. Trophic levels were then used to assign body sizes as 10^*T*_*i*_−1^ where *T*_*i*_ is the trophic level of species *i* [30], and mass-specific metabolic rates were assigned using 3/4-power scaling [23–25]. Initial abundances were drawn from a uniform distribution ranging from 0.5 to 1, ensuring that the simulation results were not artifacts of a particular set of initial conditions and that all species were initially present at ecologically significant levels. The simulation was then run for 1000 time steps using fourth-order Runge-Kutta integration with a step size of 0.01, after which time a steady state had been reached or closely approximated. At that point, any extinct species were removed and the simulation run for 1000 more time steps. In order to avoid transient dynamics, only this second run was analyzed with DEA.

The effect of food web size (10 to 50 species) and connectance (0.1 to 0.48, in increments of 0.02) on flow indirectness was examined. Because the niche model is stochastic, 250 model realizations were generated and simulated for each pair of size and connectance values.

### Dynamic Environ Approximation

In the standard Yodzis-Innes model, the amount of energy gained by a predator in a predation event is less than the amount lost by the prey. The boundary inputs and outputs required to balance the system’s energy budget are not explicitly tracked. Therefore, in order to create the conservative flow matrix required for environ analysis, producer growth was conceptualized as an input to the system, while uneaten or unassimilated food and metabolic losses were conceptualized as outputs. For each integer time step, a flow matrix consisting of the second terms of Eq 3 (with negative outflows from each compartment on the diagonal) was set and used to compute the throughflow-normalized flow matrix **G** (*g*_*ii*_ = 0, $g_{ij}=\frac{f_{ij}}{t_{j}^{out}}$, where *f*_*ij*_ is the energy flow from *j* to *i* and $t_{j}^{out}$ is the total outflow from *j*). This was then used to perform DEA with a window size of 20, which previous work indicated is typically enough to capture all relevant dynamics [14, 33]. As a large majority of simulation runs had reached or nearly reached a constant steady state, the **N** matrix was only computed for one starting time. Flow indirectness (FI) was then calculated as **N** − **G** and the mean for each web was computed. In 62 runs out of 205,000, FI values larger than 1 or less than 0 were obtained; it was concluded that the integration step size was too large for the dynamics of these runs and they were excluded from further analysis. FI values for diagonal entries, which represent cycles linking a species to itself, were taken to be 0.

All simulation and analysis code is given in Supporting Information S1 Source Code.

## Supporting Information

S1 Text Listing of variables used in CART analysis. (PDF)

S1 Source Code C++ code for food web simulation and environ analysis. (ZIP)
