# Correction: Indirect Energy Flows in Niche Model Food Webs: Effects of Size and Connectance

**DOI:** 10.1371/journal.pone.0141636

**Published:** 2015-10-21

**Authors:** Jane Shevtsov, Rosalyn Rael

There is an error in the legend for Fig 1, “Direct and indirect flows.” The complete, correct Fig 1 legend is:

**Fig 1:**
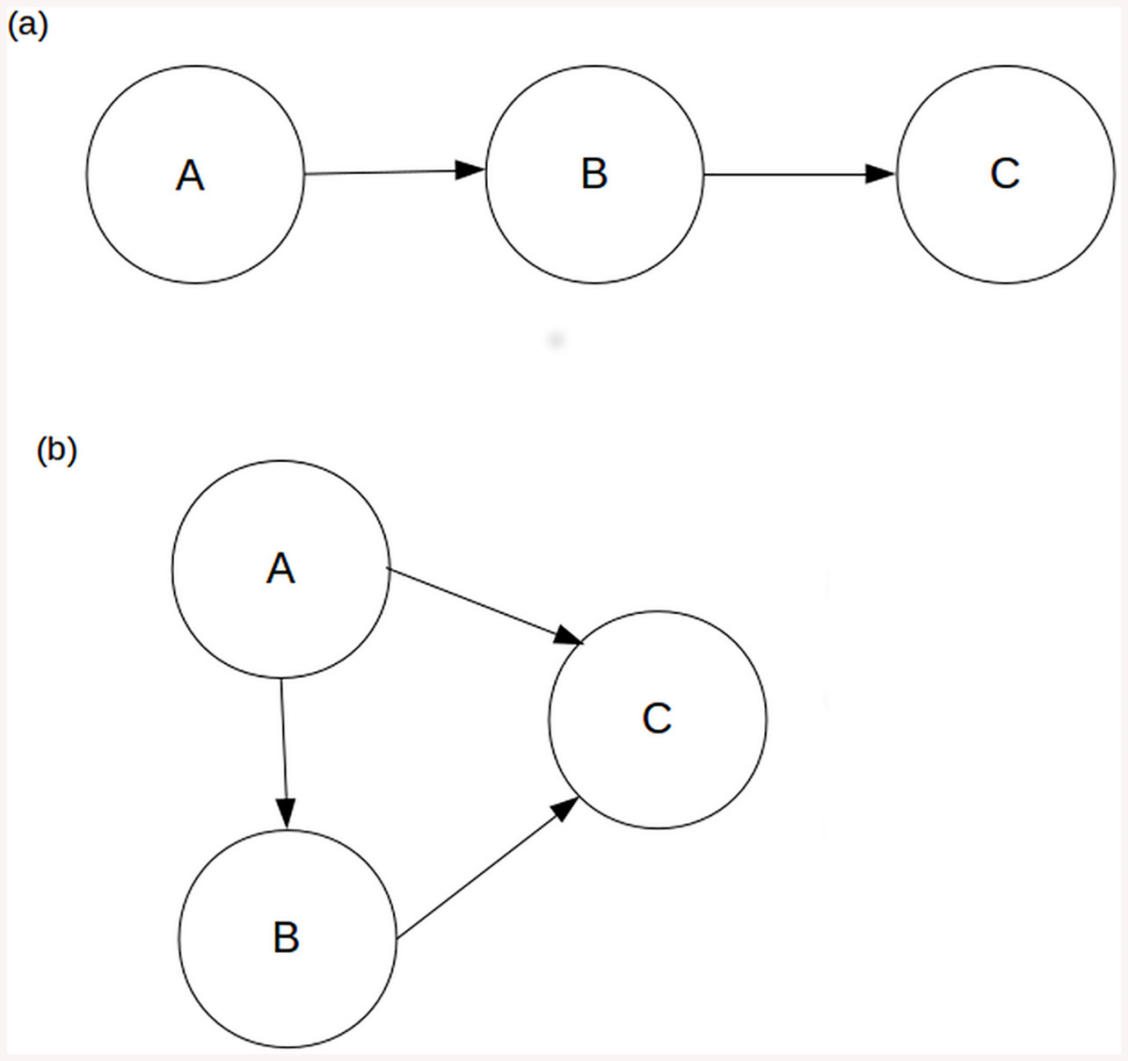
Direct and indirect flows. In the module shown in Fig 1a, species A and C are linked exclusively by indirect flows, while in the module shown in Fig 1b, they are linked by both direct and indirect flows.
