# Supplementary material for: Indirect Energy Flows in Niche Model Food Webs: Effects of Size and Connectance
Source: PLoS One. 2015 Oct 5;10(10):e0137829. doi: 10.1371/journal.pone.0137829 (PMC4593635; doi:10.1371/journal.pone.0137829)
Supplement: S1 Text — (PDF) [file pone.0137829.s001.pdf]

Supporting Information

Variables Used in CART Analysis

27 variables were used as potential predictors in the CART analysis. They included nominal and actual size and connectance, mean path length between all pairs of species, graph diameter (the largest minimum distance between two nodes), and clustering coefficient (the probability that two nodes that share a neighbor are themselves connected). Structural cycling was quantified as either dominant or maximum eigenvalue [1]. Several properties of the degree distribution of the adjacency matrix were also used as potential predictors. These were the mean, median, maximum, minimum, variance and mean absolute deviation from the median (MADAM). The same measures were applied to trophic levels. Finally, several explicitly ecological quantities were used. These were the mean generality (the number of prey a species has), mean vulnerability (number of predators), and the fraction of species at top, bottom and intermediate trophic levels.

References

[1] Borrett, Stuart R and Fath, Brian D and Patten, Bernard C (2007) Functional integration of ecological networks through pathway proliferation. Journal of Theoretical Biology, 245: 98–111
